# MultiFacTV: module detection from higher-order time series biological data

**DOI:** 10.1186/1471-2164-14-S4-S2

**Published:** 2013-10-01

**Authors:** Xutao Li, Yunming Ye, Michael Ng, Qingyao Wu

**Affiliations:** 1 Department of Computer Science, Shenzhen Graduate School, Harbin Institute of Technology, Shenzhen, 518055, China; 2 Department of Mathematics, Hong Kong Baptist University, Kowloon Tong, Hong Kong, China; 3 Shenzhen Key Laboratory of Internet Information Collaboration, Shenzhen, 518055, China

## Abstract

**Background:**

Identifying modules from time series biological data helps us understand biological functionalities of a group of proteins/genes interacting together and how responses of these proteins/genes dynamically change with respect to time. With rapid acquisition of time series biological data from different laboratories or databases, new challenges are posed for the identification task and powerful methods which are able to detect modules with integrative analysis are urgently called for. To accomplish such integrative analysis, we assemble multiple time series biological data into a higher-order form, e.g., a gene × condition × time tensor. It is interesting and useful to develop methods to identify modules from this tensor.

**Results:**

In this paper, we present MultiFacTV, a new method to find modules from higher-order time series biological data. This method employs a tensor factorization objective function where a time-related total variation regularization term is incorporated. According to factorization results, MultiFacTV extracts modules that are composed of some genes, conditions and time-points. We have performed MultiFacTV on synthetic datasets and the results have shown that MultiFacTV outperforms existing methods EDISA and Metafac. Moreover, we have applied MultiFacTV to Arabidopsis thaliana root(shoot) tissue dataset represented as a gene×condition×time tensor of size 2395 × 9 × 6(3454 × 8 × 6), to Yeast dataset and Homo sapiens dataset represented as tensors of sizes 4425 × 6 × 6 and 2920×14×9 respectively. The results have shown that MultiFacTV indeed identifies some interesting modules in these datasets, which have been validated and explained by Gene Ontology analysis with DAVID or other analysis.

**Conclusion:**

Experimental results on both synthetic datasets and real datasets show that the proposed MultiFacTV is effective in identifying modules for higher-order time series biological data. It provides, compared to traditional non-integrative analysis methods, a more comprehensive and better view on biological process since modules composed of more than two types of biological variables could be identified and analyzed.

## Background

Identification of biological modules plays a key role in bioinformatics because it can reveal interesting groups of proteins/genes having strong interactions, which may be related to some biological functionalities. In the literature, many methods have been proposed for this purpose. One popular way is to make use of clustering algorithms [1-4], which reveals module patterns by clustering proteins/genes into groups such that intragroup similarities are maximized while inter-group similarities are minimized. The performance of this type of methods relies significantly on the similarity function used during the clustering process. Due to this shortcoming, some researchers also tune to matrix factorization techniques for detecting biological modules. For example, in [5-7], singular value decomposition based methods have been studied and developed to detect modules from gene expression data. In [8-10], nonnegative matrix factorization related methods have been developed to cluster and explore biological data. Recently, CUR decomposition, a new method approximating original data matrix by selecting a set of columns and rows, has been applied to analyze microarray data and SNP data [11,12] because of its scalability and interpretability. This method may possibly be used to cluster large-scale biological data as well. However, all these methods are developed for analyzing biological data represented as matrix form, which models interactions between only two types of variables.

With rapid acquisition of biological experiments from different laboratories or studies based on different databases, many higher-order biological data representing interactions between more than two types of variables can be obtained. For instance, researchers in different laboratories may be interested in analysing gene co-expression networks under different stimulus, each of which is represented as a gene×gene matrix. Integrating these matrices results in a higher-order biological data, namely a gene×gene×stimulus tensor, and finding module patterns from such data tends to offer a better view of the underlying biological structures. Therefore powerful methods which are able to detect modules with integrative analysis are urgently called for.

In the literature, several integrative analysis methods have already been put forward. In [13], Li et al. developed a framework to find *recurrent heavy subgraphs *from multiple weighted networks represented as a 3D tensor, i.e., gene × gene × network. In the framework, a tensor objective function is proposed and solved, the solution of which helps to discovery a heavy subgraph. In [14], Omberg et al. employed higher-order singular value decomposition(HOSVD) to perform integrative analysis of multiple mircoarray data from different studies. Zhang et al. extended nonnegative matrix factorization method for exploring protein modules from multiple data sources [15]. In [16], a JointCluster algorithm was proposed to extract coherent clusters from multiple networks. However, all these methods are not suitable for analyzing time series data, which is also a task of particular importance in bioinformatics.

In this paper, we are interested in identifying biological modules from multiple time series data with integrative analysis. There are two ways to build up such data in general. One is to collect and accumulate from different time series data sources [17,18], and the other is to perform time series biological experiments under different stimulus/conditions [19,20]. The second way is usually more popular. For instance, in [20], researchers studied the time series expression profiles of genes in Arabidopsis thaliana under several abiotic stimulus; in [19], researchers studied time series gene expression of several sclerosis patients after IFN-*β *injection. Joining such data together, we can form a higher-order time series tensor, e.g., a gene×condition×time tensor. Identifying modules of genes, conditions and time-points from such tensor data could offer us a better understanding of the corresponding biological processes. For example, Supper et al. proposed EDISA algorithm by extending the 2D *iterative signature algorithm *to extract and analyze such modules [21].

We propose in this paper, MultiFacTV, a method to find modules from tensor time series data. This method employs a tensor factorization objective function and makes use of the decomposition results to identify modules. As we consider time series data, the modules are expected to be as consecutive as possible in time dimension. Therefore we incorporate a time-related regularization term of total variation into the objective function. Different from the conference version [22], we have re-derived the factorization formulas and updated the algorithm because we do not assume that input biological tensor is nonnegative in this paper. We have compared MultiFacTV with EDISA [21] and MetaFac [23] on synthetic datasets, and the results have shown that MulitFacTV outperforms the other two algorithms. In addition, we have applied MultiFacTV to Arabidopsis thaliana root(shoot) tissue dataset, Yeast dataset and Homo Sapiens dataset, and the results have shown that MultiFacTV indeed identifies some interesting biological modules, most of which have not yet been reported in our conference version. These interesting findings have also been validated and explained by using Gene Ontology analysis with DAVID or other analysis.

## Methods

### Terminologies

A tensor refers to a multidimensional array or matrix. The order of a tensor is defined to be the number of dimensions, also known as modes, of the corresponding multidimensional array. For instance, given a *n*_1 _× *n*_2 _× *n*_3 _tensor $A=\left(a_{r,s,t}\right)$, it is called a third-order tensor. The process of rearranging a tensor into a two-dimensional matrix is called unfolding. A *n*-th order tensor can be unfolded into *n *matrices in terms of each of its modes. For example, unfolding the tensor A  in terms of mode 1, mode 2 and mode 3, we obtain three matrices **A**^(1)^, **A**^(2) ^and **A**^(3) ^of sizes *n*_1 _× *n*_2_*n*_3_, *n*_2 _× *n*_1_*n*_3 _and *n*_3 _× *n*_1_*n*_2 _respectively. In this paper, we let **A**^(*p*) ^denote the unfolding matrix of tensor A  in terms of mode *p*.

Let **I**_*n*×*n *_be the *n *× *n *identity matrix. Let **M***^T ^*be the transpose of matrix **M**. Given a *n*_1 _× *n*_2 _matrix **M**, we define *vec*(**M**) to be a *n*_1_*n*_2 _× 1 vector that is obtained by stacking each column of **M**. We define shrinkage_*α*/*ρ*_(*·*) to be a shrinkage-thresholding operator for each entry of a matrix, i.e.,

$$\text{shrinkag}{\text{e}}_{\alpha /\rho }{\left(M\right)}_{i,j}=m_{i,j}-\text{min}\left(\alpha /\rho ,|m_{i,j}|\right).\frac{m_{i,j}}{\left|m_{i,j}\right|},$$

where $\frac{m_{i,j}}{\left|m_{i,j}\right|}$ should be zero when *m_i,j _*= 0.

Let ⊗ and ○ be the Kronecker product operator and outer product operator. Given a *n*-dimensional vector **x **= [*x*_1_, *x*_2_, ..., *x_n_*]*^T ^*, let **x**^+ ^= {*x_i_|x_i _>*0, 1 *≤ i ≤ n*} and **x**^- ^= {*x_i_|x_i _<*0, 1 *≤ i ≤ n*} denote the sets of its positive entries and negative entries respectively. Besides, we define $\sum {\text{x}}^{+}=\sum_{y\in {\text{x}}^{+}}y$ and $\sum {\text{x}}^{-}=\sum_{y\in {\text{x}}^{-}}y$. In this paper, max(*·*) and min(*·*) are functions used to find the maximum value and minimum value respectively.

### MultiFacTV

**Figure 1:**
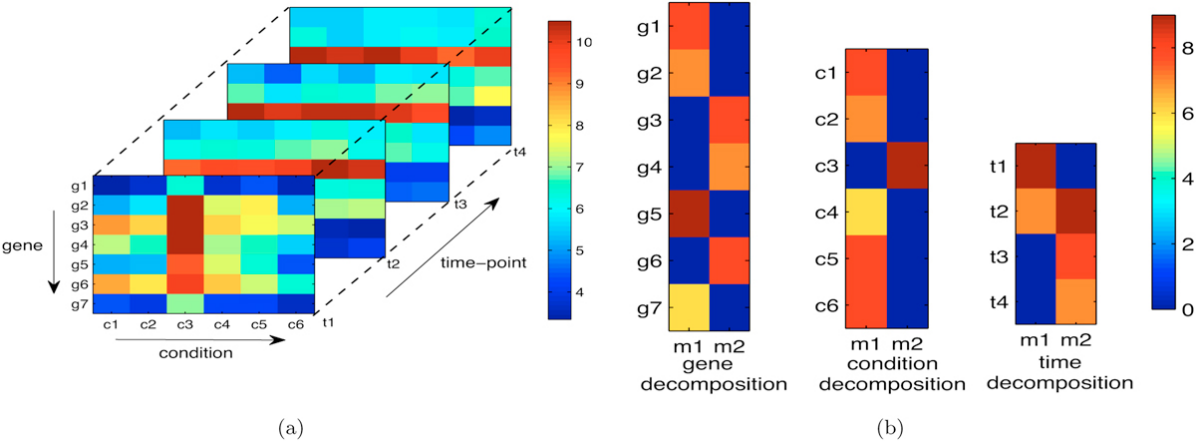
**Illustration of module detection by using tensor factorization**. (a) higher-order biological (tensor) data; (b) decomposition vectors obtained by factorizing the corresponding tensor.

Our idea to extract modules from higher-order time series biological data is using tensor factorization techniques. A higher-order time series biological data can be represented as a tensor. For example, a gene-condition-time interaction data is represented as a tensor in Figure 1(a). Factorizing this tensor with two decompositions for gene, condition and time-point respectively, we find two modules, i.e., the first module *m*_1 _= {*g*_1_, *g*_2_, *g*_5_, *g*_7_, *c*_1_, *c*_2_, *c*_4_, *c*_5_, *c*_6_, *t*_1_, *t*_2_} and the second module *m*_2 _= {*g*_3_, *g*_4_, *g*_6_, *c*_3_, *t*_2_, *t*_3_, *t*_4_}, by using a threshold (say 4) to cut off the decompositions shown as in Figure 1(b). However, we may not be able to obtain good modules merely based on traditional tensor factorization techniques because the data we are considering includes time dimension. We need to make sure the modules exist consistently in some consecutive time periods, e.g., the time-points involved in module *m*_1_/*m*_2 _are expected to be as consecutive as possible. To achieve this property, some suitable constraints must be incorporated into the factorization process. Next we will formulate the proposed MultiFacTV method.

We assume that the higher-order biological data represents interactions between three types of variables, for example gene × condition × time data. We formulate the proposed MutliFacTV based on such data in this paper. However, it is remarkable that MultiFacTV is a general framework that can be derived similarly for biological data representing interactions more than three types of variables. Suppose we consider the genomic expression profiles of *n*_1 _genes under *n*_2 _conditions over *n*_3 _time-points. The corresponding interactions can be represented as a *n*_1 _× *n*_2 _× *n*_3 _tensor $A=\left(a_{r,s,t}\right)$, where *a_r_*_,*s*,*t *_is a value recording how the gene *r *responds to the condition *s *at the time-point *t*. We note that *a_r_*_,*s*,*t *_can be a positive or negative value, i.e., the input tensor A  is not necessarily a nonnegative tensor.

Assume we would like to find *K *modules. The following objective function is proposed to decompose the tensor A  into three matrices **U**, **V **and **W**:

(1) $$\begin{array}{l} \text{min}{∥A-\overset{K}{\underset{k=1}{\sum }}u_{k}\circ \;v_{k}\circ w_{k}∥}^{2}+\alpha \overset{K}{\underset{k=1}{\sum }}||Bw_{k}|{|}_{1}\\ \text{s}\text{.t}\text{.}\;\;W\geq 0,\;\;\;\text{and}\;\;1^{T}\;\cdot \;\;w_{k}\;=\;1\;\;\text{for}\;\;k\text{ = 1,}\;\text{2, }...\text{,}\;K \end{array}$$

where **U **= [**u**_1_, **u**_2_, ..., **u***_K_*], **V **= [**v**_1_, **v**_2_, ..., **v***_K_*], **W **= [**w**_1_, **w**_2_, ..., **w***_K_*] are three decomposition matrices regarding *n*_1 _genes, *n*_2 _conditions and *n*_3 _time-points respectively; **B **is a (*n*_3 _- 1) *× n*_3 _matrix satisfying

$$b_{i,j}=\left\{\begin{array}{cc} 1 & i=j\\ -1 & i=j-1\\ 0 & \text{otherwise} \end{array}\right)$$

and α *>*0 is a regularization parameter. Clearly, $\alpha \;\overset{K}{\underset{k=1}{\sum }}||Bw_{k}|{|}_{1}$ is a total variation constraint regarding the decomposition matrix of time. With this regularization term, we can control the modules identified such that they exist consistently in some consecutive time periods. Different from the conference version [22], the decomposition matrices **U **and **V **do not have nonnegative constraints because we allow negative entries in the tensor A .

MultiFacTV seeks matrices **U**, **V **and **W **that minimize the objective function in (1). As there are three matrices unknown, we need to solve them in an iterative fashion, i.e., changing the optimization problem into three subproblems with one unknown matrix in each, and then solving them iteratively until it converges. Therefore we have three subproblems for MultiFacTV as follows.

**Subproblem 1: **Fix **V **and **W**, and solve **U **by minimizing the objective function in (1).

In this subproblem, the objective function is transferred into:

(2) $$\text{min}\;{∥A^{\left(1\right)}-UF∥}^{2}$$

where **F **= (**W **ʘ **V**)*^T^*. We have the following solution for **U**:

(3) $$U=A^{\left(1\right)}F^{T}{\left(FF^{T}\right)}^{-1}$$

**Subproblem 2: **Fix **U **and **W**, and solve **V **by minimizing the objective function in (1).

In this subproblem, the objective function is transferred into:

(4) $$\text{min}{∥A^{\left(2\right)}-VF∥}^{2}$$

where **F **= (**W **ʘ **U**)**^T^**. We have the following solution for **V**:

(5) $$V=A^{\left(2\right)}F^{T}{\left(FF^{T}\right)}^{-1}.$$

**Subproblem 3: **Fix **U **and **V**, and solve **W **by minimizing the objective function in (1).

In this subproblem, the objective function is transferred into:

(6) $$\begin{array}{l} \text{min}{∥A^{\left(3\right)}-WF∥}^{2}+\alpha \overset{k}{\underset{k=1}{\sum }}||Bw_{k}|{|}_{1}\\ \text{s}\text{.t}\text{.}\;\;\;\;1^{T}\cdot \;\;w_{k}\text{ = }1\text{ for }k=1,\;2,...\text{, }K\text{,} \end{array}$$

where **F **= (**V **ʘ **U**)*^T^*. In order to solve the matrix **W **in (6), we introduce two (*n*_3 _- 1) × *K *auxiliary matrices **P **and **Q **and adopt the strategy of Alternating Direction Method of Multipliers (ADMM) [24,25]. As a result, three updating formulas are derived and obtained (see [22] for the detailed derivation):

(7) $$vec\left(W\right)\;=\;{\left(\left(FF^{T}⊗2I_{n_{3}\times n_{3}}\right)+\left(\rho I_{K\times K}⊗\;B^{T}B\right)\right)}^{-1}\;vec\;\left(\rho B^{T}\left(P-Q/\rho \right)+2A^{\left(3\right)}F^{T}\right)$$

(8) $$P=\;\text{shinkag}{\text{e}}_{\alpha /\rho }\left(BW+Q/\rho \right)$$

(9) Q=Q+ρ(BW-P)

Here *ρ *can be any positive number and we use *ρ *= 1 in our implementation. Clearly, we need to update matrices **W**, **P **and **Q **iteratively until it converges to solve this subproblem. Note that each column of **W **must be normalized after updating as equation (7) to guarantee its constraints in (1).

Iteratively solving these three subproblems leads to a local minimum of the MultiFacTV objective function in (1) and the solutions for matrices **U**, **V **and **W **at the same time. Different from our conference version, the updating formulas for **U **and **V **in Subproblems 1 and 2 change here because we do not have nonnegative constraints on them. Next we summarize the proposed MultiFacTV method in Algorithm 1.

**Algorithm 1 **The MultiFacTV Algorithm

**Input: **a *n*_1 _× *n*_2 _× *n*_3 _tensor A , the number of modules *K*, parameter *α*, and thresholding parameters *τ*_1_, *τ*_2_, and *τ*_3_

**Output**: *K *modules stored in Ω = {Ω_1_, Ω_2_, ..., Ω*_K _*}

**Procedure**:

 1: Randomly initialize matrices **U**_(0)_, **V**_(0) _and **W**_(0)_, and set *t *= 1;

 2: Compute **U**_(*t*) _= **A**^(1)^**F***^T ^*(**FF***^T^*)^-1 ^where **F **= (**V**_(*t*-1_) ʘ **U **_(*t*-1)_)*^T^*;

 3: Compute **V**(*t*) = **A**^(2)^**F***^T ^*(**FF***^T^*)^-1 ^where **F **= (**W**_(*t*-1) _ʘ **U **_(*t*)_)*^T^*;

 4: Randomly initialize matrices **P**_(0) _and **Q**_(0)_, and set **F **= (**V**_(*t*) _ʘ **U**_(*t*)_)*^T^*, *s=*1, *ρ=*1;

 5: Iteratively update **W**_(*s*)_, **P**_(*s*)_, **Q**_(*s*) _as follows:

$$\begin{array}{l} vec\left(W_{\left(s\right)}\right)={\left(\;\left(FF^{T}⊗\;\;2I_{n3\times n3}\right)+\left(\rho I_{K\times K}⊗B^{T}B\right)\right)}^{-1}\;vec\;\left(\rho B^{T}\left(P_{\left(s-1\right)}-Q_{\left(s-1\right)}/\rho \right)+2A^{\left(3\right)}F^{T}\right),\\ P_{\left(s\right)}=\text{shinkage}{\;}_{\alpha /\rho }\left(BW_{\left(s\right)}+Q_{\left(s-1\right)}/\rho \right),\\ Q_{\left(s\right)}=Q_{\left(s-1\right)}+\rho \;\left(BW_{\left(s\right)}-P_{\left(s\right)}\right), \end{array}$$

  until it converges;

 6: Set **W**_(*t*) _= **W**_(*s*)_;

 7: If *||***U**_(*t*) _- **U**_(*t*-1)_*||*^2 ^+ ||**V**_(*t*) _- **V**_(*t*-1)_*||*^2 ^+ *||***W**_(*t*) _- **W**_(*t*-1)_*||*^2 ^*>*0.001, set *t *= *t *+ 1 and goto Step 2;

  otherwise, goto Step 8;

 8: For *k *= 1 to *K*

   Set Ω*_k _*= Ø

   $\text{If }\sum u_{k}^{+}<-\sum u_{k}^{-},\;\text{set}\;u_{k}\;=\;-u_{k};$

   For *r *= 1 to *n*_1_

    $\text{If}\;u_{r,k}>\;\;=\;0.\text{5}*\tau_{\text{1}}*\left((\text{max}\left(u_{k}^{+}\right)+\text{min}\left(u_{k}^{+}\right)\right)\text{, set }\Omega_{k}=\;\Omega_{k}\cup \left\{\text{gene }r\right\};$

   $\text{If }\sum v_{k}^{+}<-\sum v_{k}^{-},\text{ set }v_{k}=-v_{k};$

   For *s *= 1 to *n*_2_

    $\text{If }v_{s,k}>\;=\;0.\text{5}*\tau_{\text{2}}*\left((,\text{max}\;\left(v_{k}^{+}\right)+\text{min}\left(v_{k}^{+}\right)\right),\text{ set }\Omega_{k}=\;\Omega_{k}\cup \left\{\text{condition }s\right\};$

   For *t *= 1 to *n*_3_

    If *w_t,k _>*= 0.5 *∗ τ*_3 _*∗ *((max(**w***_k _*)+min(**w***_k _*)), set Ω*_k _*= Ω*_k _*∪ {time point *t*};

 9: Return Ω = {Ω_1_, Ω_2_, ..., Ω*_K_*}.

In this algorithm, we need to input a tensor and five parameters. At the beginning, the algorithm randomly initializes matrices **U**, **V **and **W **in step 1, and then it updates them iteratively from steps 2 to 7. We note that there is an inner loop in step 5 in order to update **W**. When finishing the computation of **U**, **V **and **W**, the algorithm outputs *K *modules in step 8 by cutting off each column of **U**, **V **and **W **with thresholding parameters *τ*_1_, *τ*_2_, and *τ*_3 _respectively. Since the decomposition matrices **U **and **V **are not necessarily nonnegative, the module extraction in step 8 is also different from the conference version.

## Results

In this section, we run MultiFacTV on synthetic datasets, Arabidopsis thaliana dataset, Yeast dataset and Homo sapiens dataset to test its performance and usefulness. The synthetic datasets are generated artificially and the other three real datasets can be found on http://www.ra.cs.uni-tuebingen.de/software/EDISA/downloads/index.htm.

### Results on synthetic datasets

In this experiment, we generated gene×condition×time tensor data to test the effectiveness of MultiFacTV. In the synthetic datasets, some "ground-truth" modules containing a set of genes, conditions and consecutive time intervals were generated. There were 400 genes, 400 conditions and 50 time-points. Based on the number of modules included, the datasets were categorized into four types, 3-module dataset, 5-module dataset, 8-module dataset and 10-module dataset. To test the robustness of MultiFacTV, we added different level of noise in the corresponding tensors, i.e., using 0.005, 0.01 and 0.02 as densities to add noise into the tensors respectively. Our objective was to identify the "ground-truth" modules accurately.

As for a comparison, we performed EDISA and MetaFac as well. For MetaFac and MultFacTV, we set *K *to be the number of modules in the dataset. For EDISA, the sample size was set to be 20 and the iteration number was set to be 50. The parameters *τ_g _*and *τ_c _*were turned in the interval [0,1] with 0.1 as increasing step and then the best parameter values were to produce final result. For MultiFacTV, we used *τ*_1 _= *τ*_2 _= 1.0 and *τ*_3 _= 0.75. All results were evaluated based on the Fscore and NMI (Normalized Mutual Information) by considering the discovered modules and the "ground truth" modules.

**Figure 2:**
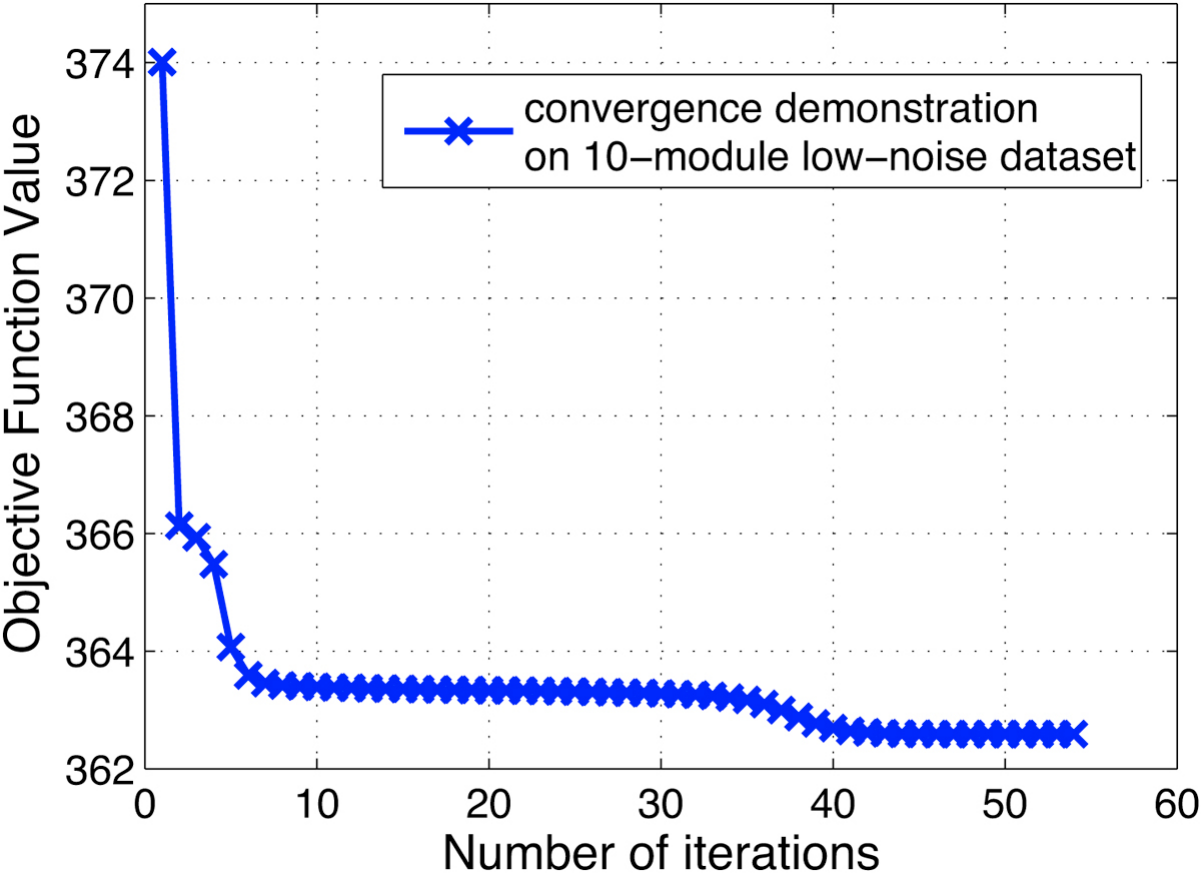
**The demonstration of convergence for a particular run of MultiFacTV on synthetic dataset**.

**Figure 3:**
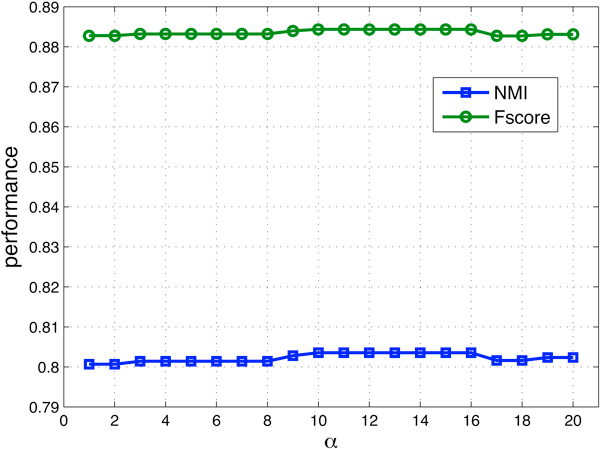
**The change of performance for MultiFacTV with respect to the tuning of parameter *α *on 10-module low noise dataset**.

Before comparing the performance of MultiFacTV and the other two algorithms, we first demonstrate the convergence of the proposed MultiFacTV and how its performance changes against the tuning of parameter *α*. In Figure 2, we show the convergence of MultiFacTV based on one synthetic dataset. We see from this figure that the objective function value is decreasing as the number of iterations increases, and after 40 iterations the change is very little and the algorithm is stopped. In Figure 3, we show how the performance of MutliFacTV changes with respect to the tuning of *α*. We see from this figure that its performance does not change significantly as parameter *α *changes from 1 to 20, and the best result is yielded when *α *= 10. Therefore we used this value for *α *in the experiments. Table 1 shows the results of EDISA, MetaFac and the proposed algorithm on these synthetic datasets. We see from the table that MultiFacTV algorithm outperforms the other two comparison algorithms.

**Table 1:** Experimental results on synthetic datasets.

Low noise level(0.005)								
	**3-module**	**dataset**	**5-module**	**dataset**	**8-module**	**dataset**	**10-module**	**dataset**
	**NMI**	**Fscore**	**NMI**	**Fscore**	**NMI**	**Fscore**	**NMI**	**Fscore**

EDISA	0.5923	0.8273	0.4997	0.7554	0.6025	0.8100	0.4512	0.6709
MetaFac	0.9117	0.9787	0.8473	0.9472	0.6181	0.7717	0.5485	0.7160
MultiFacTV	**0.9874**	**0.9982**	**0.9936**	**0.9986**	**0.8273**	**0.9140**	**0.8035**	**0.8844**

**Middle noise level(0.01)**								

	**3-module**	**dataset**	**5-module**	**dataset**	**8-module**	**dataset**	**10-module**	**dataset**
	**NMI**	**Fscore**	**NMI**	**Fscore**	**NMI**	**Fscore**	**NMI**	**Fscore**

EDISA	0.4200	0.7136	0.2907	0.6381	0.6670	0.8654	0.3142	0.6027
MetaFac	0.9312	0.9830	0.4710	0.6916	0.5444	0.7051	0.4146	0.6037
MultiFacTV	**0.9920**	**0.9987**	**0.9898**	**0.9978**	**0.8928**	**0.9552**	**0.7801**	**0.8678**

**High noise level(0.02)**								

	**3-module**	**dataset**	**5-module**	**dataset**	**8-module**	**dataset**	**10-module**	**dataset**
	**NMI**	**Fscore**	**NMI**	**Fscore**	**NMI**	**Fscore**	**NMI**	**Fscore**

EDISA	0.4493	0.7189	0.2514	0.5923	0.2055	0.4355	0.1496	0.4411
MetaFac	0.9260	0.9793	0.5318	0.6804	0.2727	0.5222	0.2479	0.4233
MultiFacTV	**0.9914**	**0.9985**	**0.9656**	**0.9898**	**0.7757**	**0.8723**	**0.6565**	**0.7747**

### Results on Arabidopsis thaliana datasets

In this experiment, the MultiFacTV was applied to Arabidopsis thaliana data to explore biological module patterns therein. The data recorded the time-series genomic expression of the root/shoot tissue in Arabidopsis thaliana when different abiotic stresses were considered. For the genomic expression data of root tissue, we constructed a gene×condition×time tensor A  of size 2395 × 9 × 6. For the genomic expression data of shoot tissue, we constructed a gene×condition×time tensor A  of size 3454 × 8 × 6. Both tensors were nonnegative. We run the MultiFacTV method with *K *= 40, *α *= 10, *τ*_1 _= *τ*_2 _= 1.0 and *τ*_3 _= 0.75 on each tensor.

Next we present some biological modules discovered from each of these tensors(data). To validate these modules, we associate them to some functional annotation terms with DAVID analysis [26]. Besides, the corresponding *p*-values are also given to demonstrate the statistical significance of these functional terms.

*Interesting genomic modules in root tissue: *Some interesting biological modules detected from root tissue by MultiFacTV are given in the following.

**Figure 4:**
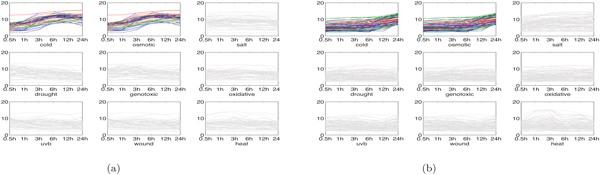
**Two cold-osmotic modules in root tissue (Both figures come from **[22]**)**. (a) genomic expression of module 1; (b) genomic expression of module 2.

**Figure 5:**
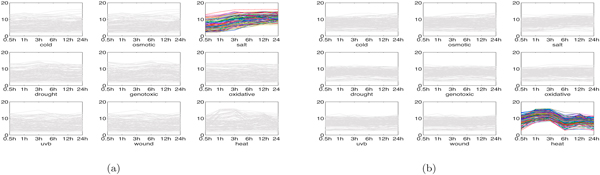
**Salt module and heat module in root tissue (Figure (b) comes from **[22]**)**. (a) genomic expression of salt module; (b) genomic expression of heat module.

**Figure 6:**
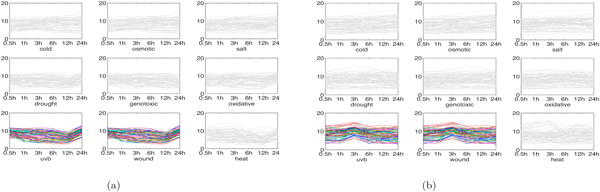
**Two Uvb-wound modules in root tissue**. (a) genomic expression of module 1; (b) genomic expression of module 2.

1. Cold-osmotic modules. In [27], it has been manifested that a large portion of the Arabidopsis genes are sensitive to cold and osmotic stress stimulus. In our results, we found several modules participating in the response to both stresses. We present two of such modules here and their genomic expression profiles are shown in Figure 4. We observe from Figures 4(a) and 4(b) there are distinct expression shapes, where the shapes for cold and osmotic conditions are quite similar. This observation indicates the genes in these two modules co-regulate under these two conditions, suggesting that Arabidopsis may not distinguish between cold and osmotic stresses. The first module is associated to functional terms like "response to water deprivation", "cold acclimation" and "response to cold" (*p*-values: 3.9 ×10^-4^, 7.1 × 10^-4 ^and 1.2 × 10^-3 ^respectively) by using DAVID, and the second module is associated to "response to osmotic stress", "response to temperature stimulus" and "response to cold" (*p*-values: 9.1 × 10^-4^, 8.9 × 10^-6 ^and 1.4 × 10^-2 ^respectively). These facts confirm that both modules play key roles in the response to cold and osmotic stresses.

2. Salt module. In Figure 5(a), we show a module detected by MultiFacTV that responds to salt stress. Apparently, this module has quite different expression shapes under salt stress compared to under the other stresses. Moreover, the terms like "response to water deprivation" and "response to salt stress" (*p*-values: 2.6 × 10^-9 ^and 5.0 × 10^-3 ^respectively) are mapped to it, which manifests this module indeed functions under salt stress.

3. Heat module. We obtained a module participating in the response to heat shock, shown as in Figure 5(b). Clearly, it has quite distinct expression shapes under heat condition. With DAVID, the genes in this module are mapped to "response to heat" and "response to temperature stimulus" (*p*-values: 1.1 × 10^-55 ^and 1.3 × 10^-43 ^respectively).

4. Uvb-wound modules. We obtained two modules responding to uvb light and wound stresses, see Figure 6. In Figure 6(a), we observe that the module 1 down-regulates slightly from 0.5h to 12h and up-regulates from 12h to 24h. This module is significant for "photosynthesis, light harvesting", "response to light stimulus" and "defense response" (*p*-values: 1.4 × 10^-8^, 2.8 × 10^-2 ^and 1.2 × 10^-2 ^respectively). It can be observed from Figure 6(b) that the module 2 has different genomic expression profiles for uvb and wound stresses in comparison with the other stresses. The module is pronounced under "response to light stimulus", "response to UV" and "response to wounding" (*p*-values: 1.4 × 10^-4^, 1.4 × 10^-4 ^and 8.7 × 10^-3^). Clearly, both modules indeed participate in the response to uvb light and wound stresses.

*Interesting genomic modules in shoot tissue: *In the following, we show two interesting genomic modules in shoot tissue output by the proposed MultiFacTV.

**Figure 7:**
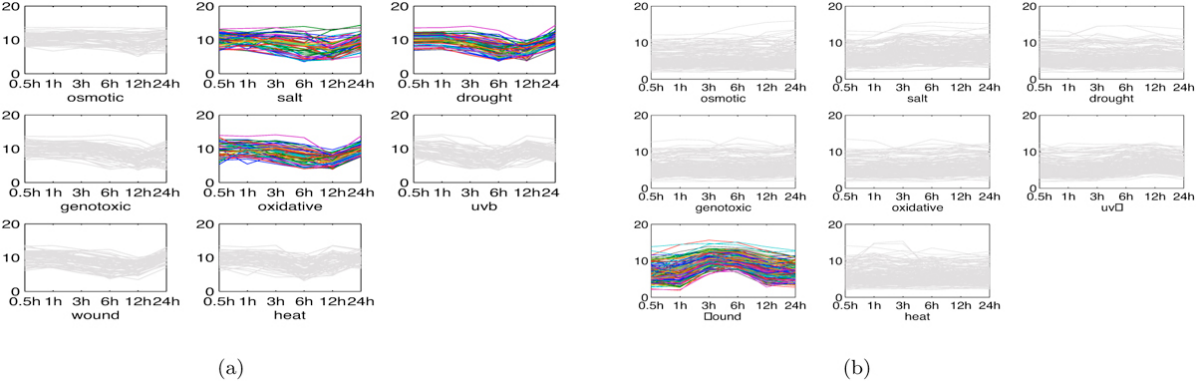
**Salt-oxidative-drought module and wound module in shoot tissue**. (a) genomic expression of salt-oxidative-drought module; (b) genomic expression of wound module.

1. Salt-oxidative-drought module. We found a module participating in the response to salt, oxidative and drought stresses, see Figure 7(a). We observe that the module has similar genomic expression profiles for salt, oxidative and drought stresses. It is annotated to functional terms like "response to salt stress", "oxidoreductase", "oxidation reduction" (*p*-values: 7.8 × 10^-2^, 3.1 × 10^-2 ^and 4.1 × 10^-2 ^respectively). This suggests that the module is significant and indeed has biological functionalities related to salt and oxidative stresses.

2. Wound module. We obtained a module participating in the response to wound stress, see Figure 7(b). It can be observed that the module first up-regulates and then down-regulates from 1h to 12h under wound stress, and its genomic expression shapes are quite different in comparison with the ones for the other stresses. By using DAVID, the module is annotated to functional terms like "defense response" and "response to wounding" (*p*-values: 2.4 × 10^-4 ^and 2.5 × 10^-2 ^respectively). This suggests the module identified by MultiFacTV indeed has wound-related biological functionalities.

### Results on yeast dataset

We performed the proposed MultiFacTV on Yeast dataset to explore interesting module patterns. This dataset recorded multiple time series genomic expression of yeast *Saccharomyces cerevisiae *regarding to different environmental changes [28]. We considered six environmental stresses in this dataset, including heat shock, 0.32mM H_2_O_2_, 1mM menadione, 2.5mM DTT(dithiothreitol), 1.5mM diamide and 1M sorbitol. Since different time-points were adopted to record the expression under different environmental stresses in the original data, we preprocessed this data by selecting 6 time-points, i.e., 10min, 20min, 30min, 40min, 60min and 80min. The missing time-point was handled by using a linear interpolation of two closest time-points available. Other missing values were replaced with the average expression value at the corresponding time-point. As a result, we constructed a gene×condition×time tensor A  of size 4425 × 6 × 6, i.e., there were 4425 genes, 6 stresses and 6 time-points. This tensor was not nonnegative because the genomic expression data included negative values. The MultiFacTV algorithm was performed with *K *= 20, *α *= 10, *τ*_1 _= *τ*_3 _= 0.75 and *τ*_2 _= 0.85.

Next we present and analyze some interesting module patterns identified by the proposed MultiFacTV.

**Figure 8:**
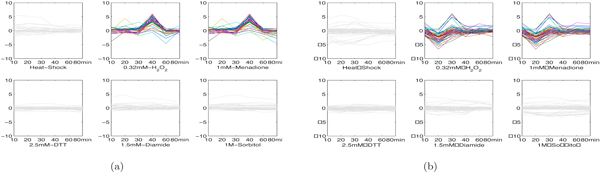
**Two H_2_O_2_-menadione modules in yeast**. (a) genomic expression of module 1; (b) genomic expression of module 2.

**Figure 9:**
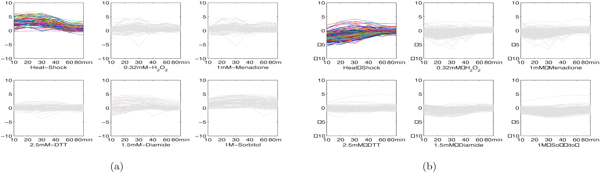
**Two heat shock modules in yeast**. (a) genomic expression of module 1; (b) genomic expression of module 2.

1. H_2_O_2_-menadione modules. In [28], it has been shown that a large portion of genes in yeast co-regulate under H_2_O_2 _stress and menadione stress despite that they are supposed to result in different reactive oxygen species. The MultiFacTV obtains similar findings and we present the genomic expression of two modules of such kind, see Figures 8(a) and 8(b). We observe that the module 1 up-regulates from 30min to 40min and down-regulates from 40min to 60min under both stresses, while the module 2 down-regulates from 10min to 20min and up-regulates from 20min to 30min. The analysis with DAVID have shown that the module 1 is functionally related to "reproduction of a single-celled organism", "mating projection tip" and "cell budding" (*p*-values: 1.9 × 10^-2^, 7.8 × 10^-2 ^and 6.1 × 10^-2 ^respectively), and the module 2 is functionally associated to "glucose catabolic process", "hexose catabolic process" and "monosaccharide catabolic process" (*p*-values: 4.2 × 10^-2^, 5.2 × 10^-2 ^and 5.8 × 10^-2 ^respectively). All these terms may be related to some biological process induced by the oxidative and reductive reactions taking place in the cells.

2. Heat shock modules. We obtained two interesting modules responding to heat shock in yeast. The genomic expression of both modules are shown as in Figures 9(a) and 9(b). We see that these two modules have opposite expression trends after heat stress where the module 1 down-regulates while the module 2 up-regulates. The analysis with DAVID have shown that the module 1 indeed takes part in the response to heat and temperature stimulus (*p*-values: 2.5 × 10^-15 ^and 3.5 × 10^-23 ^respectively). Moreover, we find this module is annotated to functional terms like "protein catabolic process" and "cellular macromolecule catabolic process" (*p*-values: 5.2 × 10^-7 ^and 2.5 × 10^-5 ^respectively). This can be interpreted by the fact that heat shock usually leads to protein unfolding [28]. The module 2 is annotated to functional terms like "ribonucleoprotein complex biogenesis" and "RNA binding" (*p*-values: 8.6 × 10^-24 ^and 7.0 × 10^-7^). This may be because the protein unfolding induces the concurrent ribonucleoprotein complex biogenesis.

### Results on Homo Sapiens dataset

We applied the proposed MultiFacTV to Homo Sapiens dataset for exploring biological modules. It was a higher-order time series dataset about genomic expression of multiple sclerosis patients after IFN-*β *injection treatment. We represented this data as a nonnegative gene×patient×time tensor of size 2920 ×14×9, i.e, there were 2920 genes, 14 patients {A, B, C, D, E, F, G, H, I, J, K, L, M, N} and 9 time-points. The MultiFacTV was performed with *τ*_1 _= *τ*_2 _= 0.5, *τ*_3 _= 0.75, *α *= 10 and *K *= 40. As a result, we found many interesting modules responding to IFN-*β *treatment similar to [21]. To exploit the usefulness of those modules from a different view, we made use of them to help us group the patients.

With the 40 modules identified, we constructed a binary matrix **M **of size 14 × 40 representing the membership of each patient to the modules, where *m_i,j _*= 1 if the *i*-th patient was associated to the *j*-th module, otherwise *m_i,j _*= 0. In such case, each of the 14 patients was represented as a 1 × 40 binary vector. Subsequently, we clustered the 14 patients by using *k*-means algorithm and the clustering results were {*A*, *B*, *C*, *D*}, {*E*, *F*, *G*, *H*}, {*J*, *K*, *L*, *M *}, {*I*, *N *}. This grouping result may suggest some differences of patients in their disease histories or progressions. We believe that this result will be beneficial to the designation of personalized medicine for the patients with multiple sclerosis [29].

## Conclusions

As more and more time series biological data are being accumulated from different laboratories or databases, identification of modules with integrative analysis become an important and urgent task. One way to accomplish such integrative analysis is assembling multiple time series biological data into a tensor form. In this paper, we have proposed the MultiFacTV method, which extends the tensor factorization objective by introducing a time-related regularization term of total variation, to detect modules from such higher-order time series biological data. We have performed the MultiFacTV method on synthetic datasets, Arabidopsis dataset, Yeast dataset and Homo sapiens dataset to test its performance. The results have shown that the proposed MultiFacTV indeed reveals some interesting module patterns. We have shown and validated these interesting findings with DAVID analysis or other analysis.

In this paper, we assume that the multiple time series genomic expression data have the same size, i.e., the same number of genes and the same number of time-points, so that they can be joined into a tensor. In some cases, the data may be in different sizes. For example, the original Yeast dataset [28] has different number of time-points for different environmental stresses. In the future, it would be interesting to extend the tensor factorization objective function of Tucker1 or Tucker2 [30] in a similar way to perform integrative module detection for such data.

## Competing interests

The authors declare that they have no competing interests.

## Authors' contributions

XL, MN and YY participated in designing the algorithm, drafting and revising the manuscript. XL participated in implementing the algorithm and performing experiments. QW participated in the discussions of experimental results. All authors have read and approved the final version of this manuscript.

## Declarations

The publication costs for this article were funded by Yunming Ye.

This article has been published as part of *BMC Genomics *Volume 14 Supplement S4, 2013: Selected articles from the IEEE International Conference on Bioinformatics and Biomedicine 2012: Genomics. The full contents of the supplement are available online at http://www.biomedcentral.com/bmcgenomics/supplements/14/S4.
